# A Process for Evaluating Adverse Environmental Impacts by Cooling-Water System Entrainment at a California Power Plant

**DOI:** 10.1100/tsw.2002.182

**Published:** 2002-05-01

**Authors:** C.P. Ehrler, J.R. Steinbeck, E.A. Laman, J.B. Hedgepeth, J.R. Skalski, D.L. Mayer

**Affiliations:** Tenera Environmental, 225 Prado Road, Suite D, San Luis Obispo, CA, 93401, USA; Columbia Basin Research, 1325 Fourth Ave., Suite 1850, Seattle, WA, 98101-2509, USA; Tenera Environmental, 100 Bush Street, Suite 850; San Francisco, CA, 94104, USA

**Keywords:** adverse environmental impact, AEI, 316(b), entrainment, cooling-water intake structure, larval mortality, fecundity hindcasting, adult equivalent loss, empirical transport model, rockfish, nearshore fish

## Abstract

A study to determine the effects of entrainment by the Diablo Canyon Power Plant (DCPP) was conducted between 1996 and 1999 as required under Section 316(b) of the Clean Water Act. The goal of this study was to present the U.S. Environmental Protection Agency (EPA) and Central Coast Regional Water Quality Control Board (CCRWQCB) with results that could be used to determine if any adverse environmental impacts (AEIs) were caused by the operation of the plant’s cooling-water intake structure (CWIS). To this end we chose, under guidance of the CCRWQCB and their entrainment technical working group, a unique approach combining three different models for estimating power plant effects: fecundity hindcasting (FH), adult equivalent loss (AEL), and the empirical transport model (ETM). Comparisons of the results from these three approaches provided us a relative measure of confidence in our estimates of effects. A total of 14 target larval fish taxa were assessed as part of the DCPP 316(b). Example results are presented here for the kelp, gopher, and black-and-yellow (KGB) rockfish complex and clinid kelpfish. Estimates of larval entrainment losses for KGB rockfish were in close agreement (FH is approximately equals to 550 adult females per year, AEL is approximately equals to 1,000 adults [male and female] per year, and ETM = larval mortality as high as 5% which could be interpreted as ca. 2,600 1 kg adult fish). The similar results from the three models provided confidence in the estimated effects for this group. Due to lack of life history information needed to parameterize the FH and AEL models, effects on clinid kelpfish could only be assessed using the ETM model. Results from this model plus ancillary information about local populations of adult kelpfish suggest that the CWIS might be causing an AEI in the vicinity of DCPP.

## INTRODUCTION

Section 316(b) of the 1972 Federal Water Pollution Control Act (Clean Water Act [CWA]) requires that "the location, design, construction, and capacity of cooling water intake structures reflect the best technology available [BTA] for minimizing adverse environmental impact [AEI]." However, the CWA does not define AEI. This has caused much concern, debate, and financial hardship for industries using water for cooling and for electric utilities in particular.

Most of the studies describing the effects of cooling-water withdrawals by electric utilities were completed in the late 1970s and early 1980s. The case of the Hudson River power plants is one of the best documented from this period[1]. After many years of debate, the case was settled out of court with the utilities contending that the intake technologies did minimize AEI even though a definition was never developed[2]. Englert and Boreman[3] stated that two points assisted in finalizing the Hudson River case: first, that converging estimates of the effects yielded increased confidence in their "realness," and second, focusing on conditional mortality instead of long-term impacts and on "defining the relative importance of each component to the analysis" was a beneficial approach.

Growing demands for new power production and a court-ordered consent decree (*Cronin v. Browner*, U.S. District Court for the southern District of New York, 93 Civ. 034), required the EPA to develop regulations for minimizing AEI caused by cooling-water intake structures (CWIS). This has kept alive the debate over the development of a clear and concise definition of AEI. Several potential definitions of AEI were presented in the proposed rules for cooling-water structures at new facilities (*Federal Register* Vol. 65, No. 155, pp. 49060–49121, August 10, 2000), but until the rule is finalized, it is not certain which, if any, of them will be used.

In an effort to evaluate the level of entrainment impact caused by the CWIS at the Pacific Gas and Electric (PG&E) Diablo Canyon Power Plant (DCPP) located in central California, we estimated entrainment effects using three mathematical models[4]. Although the plant began operation in 1985, a final 316(b) demonstration was not completed at DCPP until 1999. The DCPP 316(b) demonstration was completed under the direction of the Central Coast Regional Water Quality Control Board (CCRWQCB).

The CCRWQCB assembled a team of scientists, consultants, and industry representatives to assist their staff in the design and implementation of all aspects of the study. This Entrainment Technical Work Group (ETWG) consisted of CCRWQCB staff and their consultants, U.S. Environmental Protection Agency

(EPA) staff, PG&E and their consultants, and a consultant for an intervener group. The ETWG met every 1 to 2 months during the study to review interim reports and to discuss aspects of the study design, implementation, and analytical methods used to assess results. The CCRWQCB also convened two workshops with a larger group of state, federal, and academic fishery experts to discuss assessment approaches with the ETWG.

The ETWG determined that the 316(b) study at DCPP would only address CWIS entrainment effects because previous studies[5] had demonstrated low potential for impingement losses. The ETWG, in consultation with state, federal, and academic fishery experts, determined that using multiple approaches to assess entrainment effects would produce results that could be used to identify whether environmental impacts were adverse for a broad range of target organisms. Convergence of the results of the multiple models would provide a relative measure of confidence in our estimates of effects. However, many of the fish entrained by the DCPP CWIS were small, nearshore species with little or no reported life history information. Thus there was no way to assess impacts for many of the taxa using models that require demographic information (e.g., adult equivalent loss[6]).

In the recent 316(b) demonstration at DCPP, two demographic models, fecundity hindcasting (FH: Alec MacCall, NOAA/NMFS, Tiburon Laboratory, personal communication; [7]) and adult equivalent loss (AEL[6,8]), were used to analyze impacts on adult populations where life history information was available. A third approach, the empirical transport model (ETM[9,10]) was used on all target organisms.

Similar to the Hudson River case[1], the DCPP 316(b) was settled before a site-specific definition of AEI could be determined. Despite this, we remain hopeful that the approach we employed at DCPP could have yielded at least a site-specific definition. By combining the three assessment approaches with ancillary local adult abundance information and harvest data, we began to converge on estimates of losses due to entrainment. The next logical step would have been to determine if these losses represented an AEI.

## METHODS

### Site Description

The DCPP is a 2,200-MW, two-unit, nuclear-powered, steam-turbine plant owned and operated by PG&E. Units 1 and 2 began commercial operation in May 1985 and March 1986, respectively. Diablo Canyon is located on a coastal terrace about midway between the communities of Morro Bay and Avila Beach in San Luis Obispo County on the central coast of California (Fig. 1).

The plant's cooling-water intake is a shoreline structure consisting of bar racks, vertical traveling screens, auxiliary cooling-water systems, and four main circulating water pumps (Fig. 2). There are seven vertical traveling screens per unit that are

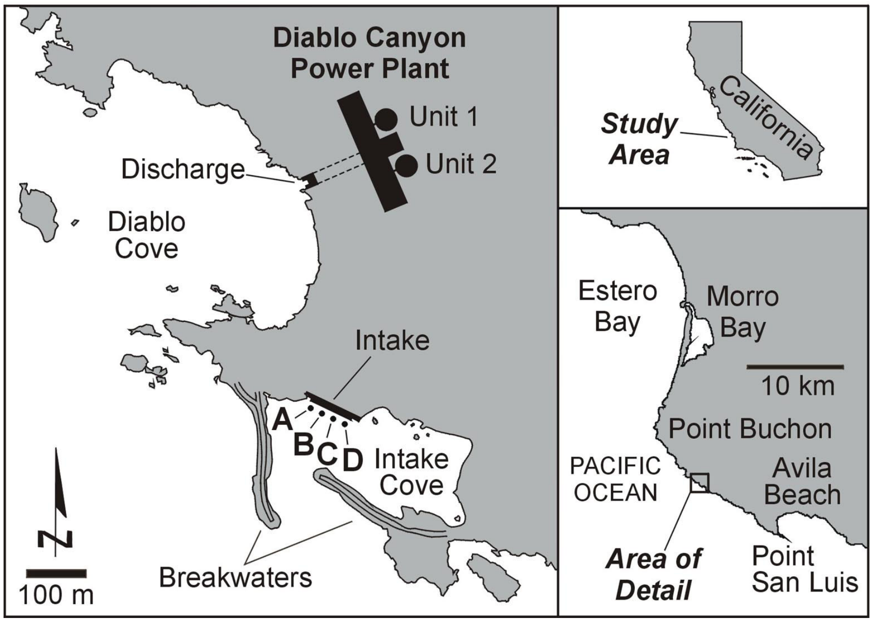

**FIGURE 1.** Diablo Canyon Power Plant, intake cove, and entrainment sampling locations (A, B, C, and D).

designed to trap and remove debris that passes through the bar racks. The screens extend from the upper deck of the intake structure to the bottom of the intake cove at a depth of approximately 10 m below sea level. The traveling screen baskets are covered with 0.95-cm mesh designed to prevent material from entering the conduits and clogging the 2.5-cm diameter condenser tubes.

The manufacturer's rated average flow rate for each of the four cooling-water pumps (CWP) at DCPP is 1,641 $m^3$/min (433,500 gallons/min)[5]. The total daily intake volume is 9.45 million $m^3$/day (2.5 billion gallons/day) when all four CWPs (two per unit) are operating. The combined flow rate of the two pumps that feed seawater to the auxiliary plant systems is 240,000 $m^3$/day (63.4 million gallons/day). The cooling-water volume withdrawn can vary daily due to changes in tidal and swell height as well as resistance caused by occlusion of the bar racks, traveling screens, or condenser tubes.

## Sampling and Processing Methods

Weekly entrainment samples were collected from a survey vessel between October 1996 and June 1999 at four permanent sampling stations (Fig. 1). Entrainment sampling took place over one 24-h period each week, with each sampling period divided into eight 3-h cycles. The four stations were sampled in random order during each cycle. Samples were collected from a boat moored to buoys located approximately 10 m from the intake and used to mark the permanent stations (Fig. 2).

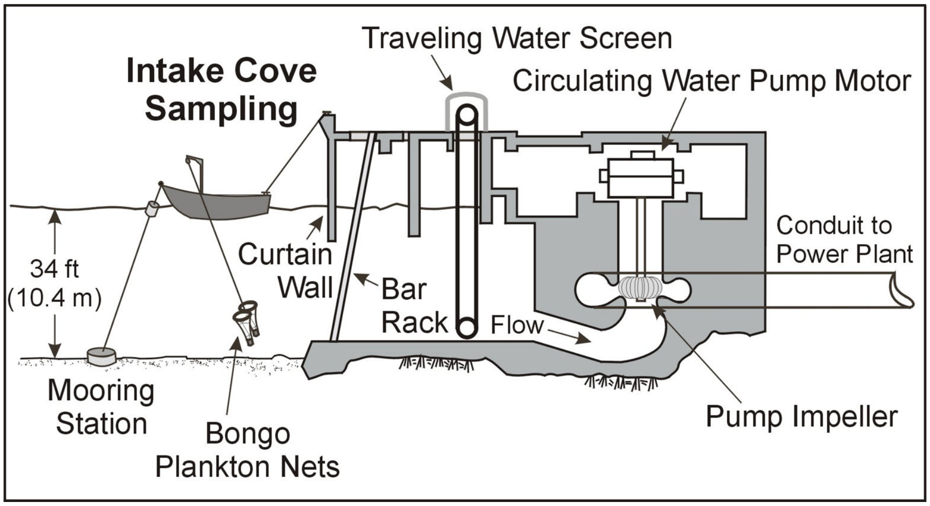

**FIGURE 2.** Cross-section view of the DCPP intake structure illustrating the location of the sampling boat and bongo nets.

A 0.71-m diameter standard CalCOFI (California Cooperative Oceanic Fisheries Investigation) style bongo frame[11,12] with two 1.8-m long nets was used for these collections. Each net mouth was fitted with a calibrated flow meter to measure the volume of water filtered. The majority of samples collected during this study employed 335 µm Nitex™ mesh nets. To achieve an adequate volume filtered, the frame and nets were fished from the surface to the bottom and then back to the surface a maximum of eight times. The net was turned at the surface and within ca. 10–30 cm of the bottom. The sinking speed of the net (0.3–0.45 m/s) was determined primarily by gravity and drag resistance on the frame and nets, while the retrieval speed (0.3 m/s) was controlled by an electric winch. The material collected in each net for all the samples collected during this study were preserved separately in either 5% buffered formalin or 70–80% ethanol. Formalin preserved samples were transferred to ethanol before laboratory processing. A total of eight subsamples (four samples) were collected per 3-h cycle for a total of 64 subsamples (32 samples) during each 24-h sampling period.

Calculation of proportional entrainment for the ETM requires an estimate of larval abundance in the source population. A survey grid centered on the DCPP intake cove was established and sampled to characterize larval abundance in the source water body (Fig. 3). The grid consisted of 64 cells set up in a symmetric eight-by eight-cell pattern. The grid extended along the coastline approximately 14 km and offshore about 3 km. The boundaries of the grid were Point Buchon to the north and Point San Luis to the south. Most areas inshore of the grid were too shallow to safely conduct boat operations in and were not sampled.

The study grid was sampled monthly from July 1997 through June 1999. Each of the 72-h study grid surveys was scheduled to bracket a 24-h entrainment survey,

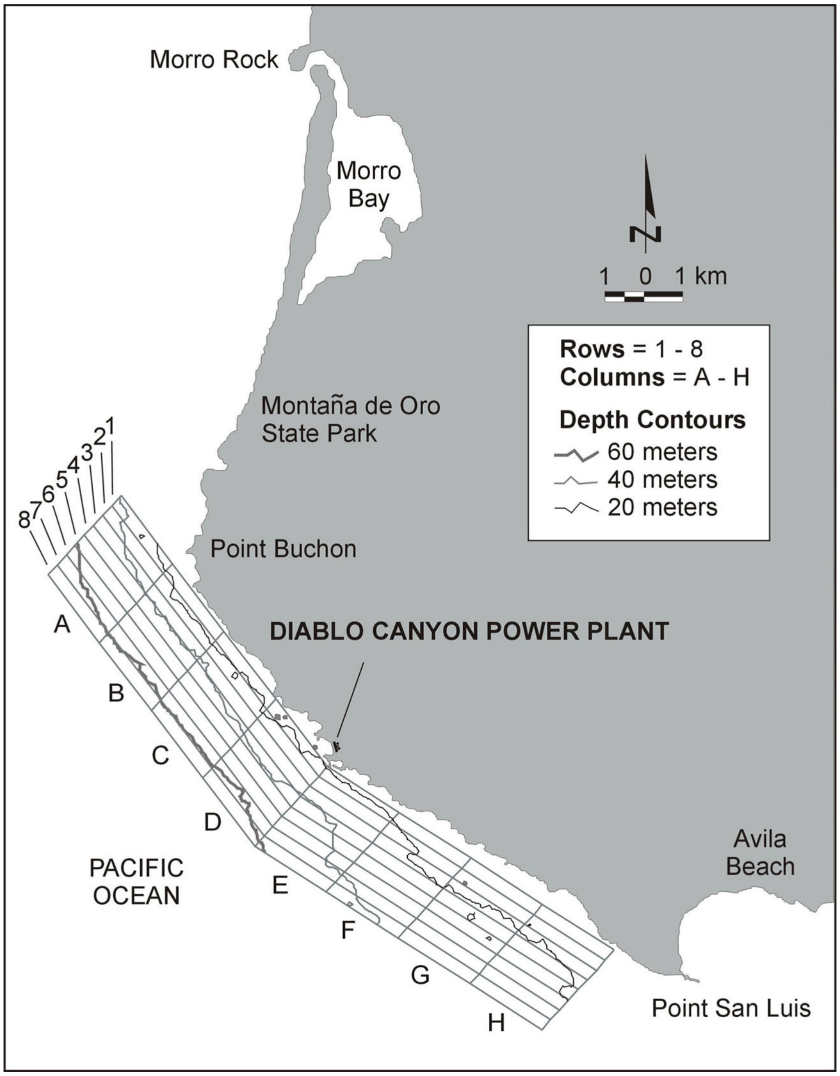

**FIGURE 3.** DCPP study grid and depth contours.

overlapping the day before and the day after entrainment sample collection. This was done to minimize temporal variation between the entrainment and study grid sampling. During each grid survey two randomly selected locations within each cell were sampled with a bongo frame using two 3.3-m long, 335-µm mesh nets. The nets were fished through the water column in an oblique manner following CalCOFI protocol[12]. The nets were lowered through the water column to within approximately 3 m of the bottom and then retrieved to the surface. Net speed through the water column was similar to that used for the entrainment sampling. A calibrated flowmeter in each net mouth measured the volume of water filtered.

In addition to the entrainment and source water samples collected during this study, data for comparison were also available from a long-term plankton sampling study conducted from 1990–1998 in the DCPP intake cove. Three samples were collected near the surface at dawn once per week by towing a 0.5-m diameter, 335-μm mesh net from the intake structure to approximately the outer end of the west breakwater. A calibrated flowmeter in the net mouth measured the volume of water filtered by the net.

Fourteen larval fish taxa and megalopal stages of all species of *Cancer* crabs were the organisms chosen by the ETWG for assessment based on ten criteria[4]. Laboratory processing consisted of removing all larval fish and *Cancer* spp. megalopae from the entrainment subsamples and from the formalin preserved grid subsamples (two per cell). A quality control program verified the removal of the target organisms from the processed samples. Larval fish and crab megalopae were identified to the lowest possible taxonomic level. A quality control program verified the identification of the larvae and megalopae. Some of the larval fish could only be identified to the familial or generic level, due to the fact that the larval stages of many fish are poorly known or undescribed.

Notochord length of most individuals of the target fish taxa was measured in the laboratory using a computer imaging system. The average length of each fish taxon per entrainment survey was used with information on larval growth found in the scientific literature to estimate the number of days the larvae had been in the plankton before being entrained.

## Ancillary Field Studies

Adult and juvenile fish populations were counted along permanent benthic subtidal transects in the vicinity of DCPP as part of the plant's Receiving Water Monitoring Program[13]. All fish observed by SCUBA divers within 2 m of either side and 1 m above the 50-m-long transect line were identified and logged onto datasheets. Two divers swam each transect but from opposite directions, with all fish being identified to the species level whenever possible. The resulting survey data were the combined species counts for both divers, divided by two, yielding an average count per 50-m transect. One area sampled by this method was located approximately 700 m to the south of the intake cove, in an area not influenced by the plant's thermal plume. The three transects in this control area range from approximately 3–10 m in depth.

## Analytical Methods

The density of the fish in the entrainment samples was used to estimate the total annual entrainment of each larval taxon $(E_T)$. A daily entrainment estimate (number of organisms/m$^3$) and its variance were calculated for each 24-h entrainment survey[7]. An estimate of the number entrained during the survey was determined by multiplying the density of each taxon by the intake water flow measured during the survey. A 100% mortality was assumed for all entrained organisms. Entrainment estimates for the period between surveys (usually 7 days) were determined by summing the product of the entrainment estimate and the daily intake volumes for the survey period. These estimates and their associated variances were then summed to obtain estimates of annual entrainment and variance using the following formulae:

$$E_T = \sum_{i=1}^{52} \left( \frac{V_i}{v_i} \right) \cdot E_i$$

and

$$Var(E_T) = \sum_{i=1}^{52} \left( \frac{V_i}{v_i} \right)^2 \cdot Var(E_i)$$

where $v_i$ is the intake volume on the survey day of the $i^{th}$ survey period ($i$ = 1,…,52), $V_i$ is the total intake volume for the $i^{th}$ survey period ($i$ = 1,…,52), and $E_i$ is the estimate of daily entrainment during the entrainment survey of the $i^{th}$ survey period.

The estimate of annual entrainment at DCPP was adjusted to better represent long-term trends for each taxon by using the longer-term intake cove plankton tow data set. These data were used to provide an index of annual trends in larval abundance for the period 1990–1998. The estimated total annual entrainment was multiplied by the quotient of the average index value from the intake cove plankton tows (1990–1998) and the index value from the surface tows during the $i^{th}$ year, thus adjusting annual DCPP entrainment by the annualized long-term average.

$$E_{adj-T} = E_T \cdot \left( \frac{\bar{I}}{I_i} \right)$$

and

$$Var(E_{Adj-T}) = Var(E_T) \cdot \left( \frac{\bar{I}}{I_i} \right)^2$$

where $E_{Adj-T}$ is the adjusted estimate of total annual entrainment adjusted to the long-term average for 1990–1998, $I_i$ is the index value from intake cove plankton tows in the $i^{th}$ year, and $\bar{I}$ is the average index value from intake cove surface tows, 1990–1998. The variance of $E_{adj-T}$ does not include the between-day, within-stratum variance, interannual variance, nor the variance associated with the indices used in the adjustment. So the actual variance is higher than what would be calculated by the above formula.

The fecundity hindcast (FH) model estimates the amount of potential female reproductive output eliminated using entrainment losses combined with estimates of female fecundity and demography (A. MacCall, NOAA/NMFS, personal communication; [7]). The number of larvae entrained by DCPP was used, along with mortality schedules from the egg stage up to the age at entrainment, to hindcast the number of females whose reproductive output could have been effectively removed from the population. This method has the advantage of needing to estimate survivorship over only a relatively short time period (i.e., egg to age at entrainment). To be extrapolated to adult losses, however, FH does require age-specific mortality rates and total lifetime fecundities that are largely unreported for species affected at DCPP. In addition, adult population estimates, typically unavailable for unfished taxa, are required to estimate population-level effects.

Estimates of the annual rate of entrainment for larval fish and subsequent FH and AEL calculations were determined for the following two analysis periods:

Period 1 – October 1996 through September 1997,
Period 2 – October 1997 through September 1998.

The plankton samples collected at the surface in the DCPP intake cove were analyzed only for the months of December through June, as this was the peak period of larval fish abundance for most of the species in this area. These data were used to estimate the long-term average abundance of each taxon that was then used to adjust the estimated annual number of larvae entrained.

The estimated total annual entrainment of each taxon $(E_T)$ was used to estimate the number of breeding females whose fecundity was potentially lost using the following formula:

$$FH = \frac{1}{F_T} \sum_{i=1}^{w} \frac{E_{Ti}}{S_i}$$

where $F_T$ is the average total lifetime fecundity for females, equivalent to the average number of eggs spawned per female over their reproductive years, $w$ is the number of weeks the larvae are vulnerable to entrainment, $E_{Ti}$ is the estimated total entrainment for the $i^{th}$ weekly survey period ($i = 1,\ldots,w$), and $S_i$ is the survival rate from the fertilized eggs to larvae of the stage present in the $i^{th}$ weekly survey period.

This equation was based on the simple case of a single synchronized spawning for a given taxon. For most taxa with overlapping or continuous spawning, larval abundance would have to be specified by week and age class. At DCPP, we used the mean size of the larvae entrained to estimate a representative larval age using daily growth rates, and then estimated a survival rate to that age. The age of the average-sized larvae in the entrainment samples was determined from length measurements and growth rates available from the scientific literature.

Assuming average rates of survival were the same between years, the adjusted annual entrainment ($E_{adj-T}$) was used in the FH approach, using the following formula:

$$FH = \frac{E_{adj-T}}{\prod_{j=1}^{n} S_j \cdot F_T}$$

where $S_j$ is the age specific survival of eggs and larvae for the $j^{th}$ age class ($j = 1,\ldots,n$), and $F_T$ is the expected number of eggs produced in a reproductive lifetime.

The expected total lifetime fecundity was approximated by the equation:

$$F_T = (\text{average eggs/year}) \cdot (\text{average number of reproductive years}).$$

The midpoint between the ages of maturation and longevity was used as the average number of reproductive years. This was based on an assumption of linear survivorship (uniform survival) between the ages of maturation and longevity. It was assumed that for exploited species, such as northern anchovy and Pacific sardine, the expected number of years of reproductive life could be less, so the estimated longevity was based on the oldest individuals caught in the fishery.

The variance of FH was approximated using the Delta method[14] in the following formula:

$$Var(FH) = FH^2\left(CV^2(E_{adj-T}) + \sum_{j=1}^{n} CV^2(S_j) + CV^2(F) + \left(\frac{Var(A_L) + Var(A_M)}{(A_L - A_M)^2}\right)\right)$$

where $CV(E_{adj-T})$ is the coefficient of variation of the adjusted entrainment estimate, $CV(S_j)$ is the CV of the estimated survival of eggs and larvae up to entrainment, $CV(F)$ is the CV of the estimated average annual fecundity, $A_M$ is the age at maturation, and $A_L$ is the age at maturity.

The following additional assumptions were made for the calculation of FH at the DCPP:

- Values of parameters from the scientific literature represent the population parameters for the years and location of this study and are constant for the population of inference;
- Reported values of egg mass are lifetime averages to calculate an unbiased estimate of lifetime fecundity;
- Reproductive life expectancy can be accurately calculated by assuming that time of death is uniformly distributed between age at maturation and age of longevity;
- Egg and larval survival rates are constant over time;
- No population reserve or compensation counters the entrainment mortality;
- The loss of the reproductive potential of one female is equivalent to the loss of an adult female; and
- A CV of 30% was assumed when no estimates of variance were available from the literature.

The AEL model estimates the loss of an equivalent number of adults (male, female, or both) based on the estimated number of entrained larvae and species-specific mortality schedules[6,8]. Survival estimates from the age of entrainment to adulthood are required for these calculations. These age-specific survivorship rates are generally not well known, except for the adults of some commercial species. For species where age-specific survival rates from larvae to adults have been estimated, AEL was calculated based on the average age of the larvae entrained. This age was determined as described for FH.

To calculate two annual estimates of larval mortality from the ETM, the monthly grid and the paired entrainment surveys were divided into the following two analysis periods:

Period 3 – July 1997 through June 1998,
Period 4 – July 1998 through June 1999.

Survivorship to adulthood (recruitment) was separated into several age stages, and AEL was calculated using the entrainment estimates adjusted to the long-term average using the following formula:

$$AEL = E_{adj-T} \prod_{j=1}^{n} S_j$$

where $n$ is the number of age classes from entrainment to recruitment, and $S_j$ is the survival rate from the beginning to end of the $j^{th}$ age class.

The variance of AEL was estimated using a Taylor series approximation (Delta method[13]) as follows:

$$Var(AEL) = AEL^2 \left( CV^2(E_{adj-T}) + \sum_{j=1}^{n} CV^2(S_j) \right)$$

In cases where survival estimates from larval entrainment to adulthood were unavailable, the fecundity hindcasting estimates could be generated as $AEL \equiv 2FH$. This treatment assumes that two animals would survive to the age to generate the average number of eggs produced in a lifespan, calculated as follows:

$$S_{\text{larvae to adult}} = \frac{2}{(S_{\text{egg + larvae}}) \cdot (\text{Fecundity}_{\text{lifetime}})}$$

where both AEL and FH can be calculated independently they offer an indication of the confidence in the accuracy of the estimate.

The following assumptions were made for the calculation of AEL:

- Literature-based life history parameters represent the fish populations during the years and at the location of the DCPP study;

- If survivorship values from the literature are limited to a single observation, they are assumed constant over time or representative of the mean;
- Survival rates used in the calculation represent the life stages of fish in the DCPP area;
- No population reserve or compensation counters the entrainment mortality; and
- A CV of 30% was assumed when no estimates of variance were available from the literature.

In some instances, survival rates were not available for the individual target species, but values for similar species were found. In these instances, an additional assumption was made for both FH and AEL:

- survival values for both species were the same.

The ETM was used to generate an estimate of the probability of larval mortality caused by entrainment ($P_M$). This model uses an estimate of the daily entrainment mortality (proportional entrainment, or PE) for each taxon based on each monthly survey. Such mortality has been referred to as conditional mortality[15]. Conditional mortality was calculated by compounding daily survival for the estimated duration that larvae would be susceptible to entrainment. The adjusted entrainment values used in the FH and AEL models were not used in the ETM results because this calculation relies on a PE ratio that uses larval abundance values from the paired entrainment and study grid surveys.

The general equation to estimate the $i^{th}$ day's PE values is:

$$PE_i = \frac{N_{Ei}}{N_{Gi}}$$

where $N_E$ is an estimate of the number of larvae entrained and $N_G$ is the estimate of the number of larvae in the study grid. To estimate the PE values, a daily entrainment estimate was paired with a corresponding estimate for the study grid survey collected over 72 h. $N_G$ was calculated using the following formula:

$$N_{Gi} = \sum_{k=1}^{64} A_{Gk} \cdot D_k \rho_{ik}$$

where $A_{Gk}$ is the area of grid cell $k$, $D_k$ is the average depth of the $k^{th}$ grid cell, and $\rho_{ik}$ is the density (#/m$^3$) of larvae in the $k^{th}$ grid cell during survey $i$.

The area inshore of study grid row 1 was too shallow to safely collect samples (Fig. 3). Since adults of many of the taxa entrained in high numbers at DCPP were likely to reside in these areas, we developed a method to include the unsampled areas in the estimates of PE[7]. The volumes of inshore areas were estimated and multiplied by the larval density in the adjacent cell to yield an estimated number of larvae in the unsampled area. The exceptions to this adjustment were cells A1, D1, and E1. Cell A1 was further offshore than the other row 1 cells due to a bend in the coastline at Point Buchon, so no adjustment was made for this cell. Cells D1 and E1 were directly off of the DCPP intake cove, so the ETWG decided that the number of larvae in the area between the grid and the intake structure would be best represented by the entrained density of each larval taxon.

The boundaries of each taxon's population could range from local (a portion of the grid) to regional (i.e., fishery management units). Boreman et al.[10] point out that if any members of the population were located outside of the area studied (the study grid at DCPP), then the ETM would overestimate the conditional[15] entrainment mortality for the entire population. The fraction of the larvae being entrained from the population of inference on a given day is then the product

$$(PE_i)\cdot(P_S)$$

where

$$P_S = \frac{N_G}{N_P};$$

the proportion of the larval population of inference $(N_P)$ that is represented by the larval population within the study grid $(N_G)$. The "proportion of the parental stock"[15], or $P_S$, can also be calculated using an estimate of the adult population in the study area. Assuming that the distribution in the larger area is uniform, the value of $P_S$ could be approximated as a ratio based on the size of the two areas. At DCPP, $P_S$ was estimated using the distance the larvae could have traveled based on the number of days it was subject to entrainment and the current velocities and patterns measured during that period. Measurements were collected at a single current meter suspended at a depth of ca. 6 m, approximately 1 km from shore. For taxa dispersed throughout the grid, both alongshore and onshore current was used in $P_S$ calculations as

$$P_S = \frac{A_G}{A_P}$$

where $A_G$ is the area of the grid and $A_P$ is the area of the population calculated from the alongshore and onshore current excursions. For taxa whose larvae were concentrated in the nearshore portions of the grid, $P_S$ was calculated as

$$P_S = \frac{L_G}{L_P}$$

where $L_G$ is the length of the grid and $L_P$ is the estimated alongshore current movement through the grid which estimates the population at risk.

The daily conditional survival is the value 1 - $PE_i$. An estimate of the larval population surviving entrainment during the $i^{th}$ survey period was generated by applying the number of days the larvae are subject to entrainment ($[1 - PE_i]^{days}$). In an attempt to provide a relevant range of survivorship estimates, the number of days that the larvae were subject to entrainment was calculated using both the average and maximum larval ages at entrainment. This provided both an average and minimum (maximum exposure to entrainment mortality) estimate of survivorship.

The monthly estimates of PE were weighted by the monthly survey fraction ($f_i$) of the source water population at risk. This was obtained from the monthly fraction of the total annual entrainment for the source water survey periods. The weighted estimates of survivorship for each survey period was then summed to provide a final estimate of $P_M$ using the following formula:

$$P_m = 1 - \sum_{i=1}^{12} f_i \cdot \left(1 - PE_i \cdot P_s\right)^{\text{days}}$$

The following assumptions were made in the $P_m$ estimations:

- Larval lengths and growth rates accurately estimate larval duration for the taxa studied;
- The estimates of conditional PE are constant within monthly survey periods;
- The monthly estimates of larval abundance represent a proportion of total annual larval production during that month; and
- $P_S$ accurately characterizes the fraction of the population of inference represented by the sampling grid.

Our intent in using three approaches to estimate the effects of larval entrainment at DCPP (i.e., FH, AEL, and ETM) was to provide several methods for determining the magnitude and quality of resulting population level impacts and as an aid to determining what constituted an AEI. While it is true that none of the three approaches is completely independent of the others, their combination still allowed us to estimate possible effects using three different methods of calculation.

## RESULTS AND DISCUSSION

There were 169,440 larval fish identified and enumerated from the processed samples (Table 1). They represented a total of 193 different taxonomic categories, ranging from the ordinal (6 taxa), family (28 taxa), genus (30 taxa), and species level (129 species). We also had a category for unidentifiable or damaged larvae and also larval fragments. From the different categories, the ETWG chose 14 fish taxa for detailed assessment using FH, AEL, and ETM.

**TABLE 1**
**Collection Period, Number of Subsamples Laboratory Processed, and Number of Larval Fish Found During the DCPP 316(b) Demonstration**

| | Sample Collection Dates | # Subsamples Processed | # Larval Fish in Subsamples Processed |
|---|---|---|---|
| Entrainment samples | Oct. 1996–June 1999 | 4,693 | 98,593 |
| Study grid samples | July 1997–June 1999 | 3,163 | 43,785 |
| Intake cove surface tows | 1990–1998 | 660 | 27,062 |

We present results for two of these taxa as a demonstration of our assessment approach using three models. Our first example is a grouping of rockfish that we nominally refer to as the kelp, gopher, and black-and-yellow rockfish (KGB) complex, and our second example is a grouping of clinid kelpfish. These two were selected for presentation here due to their high abundance in entrainment samples and because they represented varying levels of available life history information. A more detailed presentation of the results of these and the other 12 taxa can be found in the final DCPP 316(b) demonstration report[4].

## KGB Rockfish Complex

Rockfish (*Sebastes* spp.) comprise a large marine commercial and recreational fishery along the California coast and are caught from nearshore coastal habitats out onto the continental shelf and slope. Lea et al.[16] report that there are 59 species of *Sebastes* in the coastal waters of California. Although *Sebastes* are an economically important genus, larval, juvenile, and adult life history parameters are not well known for many of the species in the group.

Larval *Sebastes* are very difficult to visually identify to the species level[17,18,19,20,21,22]. Perhaps 5 or 6 of the 59 rockfish species expected to occur in the vicinity of DCPP can be identified at the early larval stage to the species level[22]: aurora rockfish (*S. aurora*), shortbelly rockfish (*S. jordani*), cowcod (*S. levis*), blue rockfish (*S. mystinus*), bocaccio (*S. paucispinis*), and stripetail rockfish (*S. saxicola*). We placed the other larval *Sebastes* into one of eight broad subgeneric groupings based on larval pigment patterns[22,23]. The most abundant *Sebastes* pigment group collected in the DCPP plankton samples was the nominal KGB complex. Based on available descriptions of larvae from identified females, species in the KGB complex (Table 2) have a common pigment pattern that distinguish them from the other larval rockfish occurring in the DCPP vicinity. Genetic analysis of a subset of larvae verified the visual identification of the KGB complex in the DCPP samples[24].

Age at maturation is approximately 5 years, and longevity is about 15 years for the species in the KGB complex[16,25,26,27, R. Larson, San Francisco State University, personal communication]. KGB rockfish are generally thought to spawn

**TABLE 2**
**Larval *Sebastes* Species Assigned to the KGB Complex**

| | |
|---|---|
| *Sebastes atrovirens* | Kelp |
| *S. auriculatus* | Brown |
| *S. carnatus* | Gopher |
| *S. caurinus* | Copper |
| *S. chrysomelas* | Black-and-yellow |
| *S. dalli* | Calico |
| *S. maliger* | Quillback |
| *S. nebulosus* | China |
| *S. rastrelliger* | Grass |
| *S. semicinctus* | Halfbanded |

once per year, with an estimated average annual fecundity of 213,158 eggs per female[28,29,30]. Female rockfish are viviparous with internal fertilization[31] and internal development of the larvae[27]. Newly released larval *Sebastes* can reside in the plankton for a period of 1 to 3 months[32,33,34].

Presence of KGB larvae in our samples was seasonal (Fig. 4a). Using estimates of weekly entrainment densities, the estimated numbers of KGB rockfish complex larvae entrained annually for the two periods, adjusted to the long-term average intake cove surface plankton tow index, were

October 1996 through September 1997 – 275,000,000 (SE = 24,700,000) larvae, and

October 1997 through September 1998 – 222,000,000 (SE = 28,900,000) larvae.

The FH calculations require estimates of the mortality rate and age at entrainment in addition to the estimated number of larvae entrained. The only mortality rate estimate available for very young larval rockfish is 0.14/day for blue rockfish (M. Yoklavich, NOAA/NMFS/PFEG, unpublished data). Despite the fact that blue rockfish are not included in the KGB complex, this value was presumed to be representative of the genus and used in FH calculations. It was estimated that the average age of entrained KGB complex larvae at DCPP was 6.2 days based on the mean length of the larvae in this group (4.2 mm) and an estimate of the daily larval growth rate from brown rockfish of 0.14 mm/day[30,31]. Using these values in FH calculations, the estimated

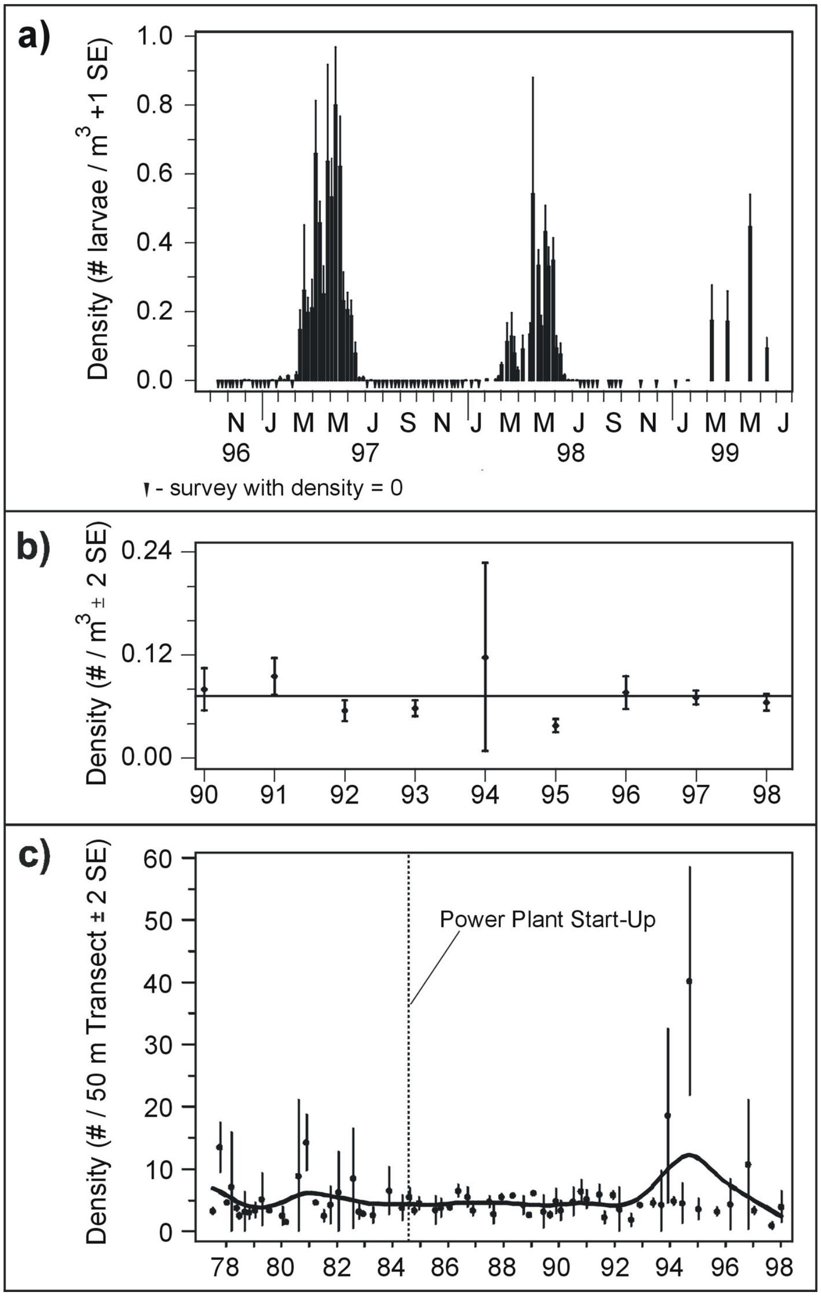

**FIGURE 4. (a)** Weekly mean density of larval KGB rockfish (#/m$^3$ + 1SE) at the DCPP intake. **(b)** Annual mean density ± 2 SE of larval KGB rockfish (vertical lines) and grand mean density for all years combined (horizontal line) for the intake cove surface plankton tows. **(c)** Mean density of juvenile and adult KGB rockfish (#/50 m transect ± 2 SE) estimated from SCUBA surveys in an area 700 m south of the DCPP intake cove. Spline smoothing algorithm used to draw the curve through the points.

number of adult female KGB rockfish whose reproductive output was potentially lost due to larval entrainment was 617 adult females for the period 1996–1997 and 497 adult females for the 1997–1998 period.

The AEL model requires survivorship estimates from the time of larval entrainment through adulthood. No estimates of KGB complex larval, juvenile, or adult survivorship were available, but survivorship for these life stages of blue rockfish had been described[22]. Early blue rockfish mortality estimates through year one were provided by M. Yoklavich (NOAA/NMFS/PFEG, Pacific Grove, CA., personal communication) and annual instantaneous mortality was assumed as 0.2/year after 1 year (Table 3). Using these survival values, the estimated number of adult equivalents (male and female) lost due to entrainment and based on the adjusted annual larval entrainment was 1,120 for the 1996–1997 period and 905 for the 1997–1998 period.

The monthly PE estimates used in calculating ETM for KGB larvae ranged from 0 to a maximum of 0.587 ± 0.297 (± 1 SE (PE)) for the 2 years studied. The highest value was calculated for March 1998, a period of peak parturition for many species in the KGB complex[33]. Due to the wide distribution of the KGB larvae throughout the grid, $P_S$ and $P_M$ were calculated using both alongshore and onshore current movements as well as average maximum estimates of larval duration. The values of $P_M$ varied from a low of 0.005 to a maximum of 0.05 depending on larval duration and current speed and direction.

Additional larval and adult abundance information collected in the vicinity of the DCPP implies a low entrainment impact on KGB rockfish complex larvae. The annual

**TABLE 3**
**3-Year Survival for the KGB Rockfish Complex Based on Blue Rockfish Data**

| Day (start) | Day (end) | Instantaneous Natural Mortality (*Z*) | Survival ($\hat{S}$) |
|---|---|---|---|
| 0 | 6.21 | 0.14 | 0.419 |
| 6.21 | 20 | 0.14 | 0.145 |
| 20 | 60 | 0.08 | 0.041 |
| 60 | 180 | 0.04 | 0.008 |
| 180 | 365 | 0.0112 | 0.126 |
| 365 | 1,095 | 0.0006 | 0.670 |

*Note:* Survival was estimated from release as $\hat{S} = e^{(-Z)(Day(end)-Day(start))}$. Daily instantaneous mortality rates (*Z*) up to 1 year of blue rockfish, *S. mystinus*, larvae that were used to calculate KGB larval survivorship were provided by M. Yoklavich (NOAA/NMFS/PFEG, Pacific Grove, CA, personal communication). Annual instantaneous mortality was assumed as 0.2/year after 1 year. Average age of entrainment was estimated as 6.21 days based on average size at entrainment and a growth rate of 0.14 mm/day[31].

mean density of KGB complex larvae in the DCPP intake cove plankton tows appears similar among years (Fig. 4b). In addition, abundance data from a combination of juvenile and adult KGB rockfish observed by SCUBA divers along permanent transects between 1978 and 1998 in an area 700 m south of the intake cove showed much intra- and interyear variation but no apparent declines in abundance over time (Fig. 4c).

Catch data from the port of Morro Bay (reported in the Pacific States Marine Fishery Council's online Pacific Coast Fisheries Information Network database were also used to provide some context for interpreting results from the three models. KGB rockfish were mainly landed as part of the live-fish fishery, and had an average price per kilogram of $7.65 in 1999 (PacFIN database). Assuming an average weight of 1 kg for a 3-year-old KGB rockfish, 100% catchability of the adult equivalents, and no compensatory mortality, the annual average estimate of 977 KGB rockfish translate to a value of about $7,500. The estimate of $P_M$ from this study for the area fished from Port San Luis (south of DCPP) to Morro Bay was between 4 and 5%. Based on the dollar value for KGB landings at Morro Bay in 1999, the proportional reduction caused by entrainment translated to a value of about $20,000 or about 2,600 1-kg adult rockfish.

The results of the three impact assessment approaches, in conjunction with additional adult abundance data, show that KGB rockfish in the vicinity of DCPP are not adversely impacted by power plant entrainment. The close concurrence of the three model results (i.e., FH - ca. 550 adult females annually; AEL - ca. 1,000 adults annually [500 adult females] worth approximately $7,500; and ETM ca. 5% or $20,000 of the local catch) provides us high confidence in our results and the conclusion that potential impacts are relatively small. Combining these results with the adult fish observations indicating a fairly stable population size confirms the conclusion of no AEI for KGB rockfish.

## Clinid Kelpfish

There are four species of adult clinid kelpfish in the DCPP area, three species of *Gibbonsia* and the giant kelpfish *Heterostichus rostratus*. The *Gibbonsia* larvae collected at the DCPP were not identifiable to the species level, so they were analyzed as a group (*Gibbonsia* spp.); *H. rostratus* were uncommon in the samples.

Very little information is available about the adult, juvenile, or larval stages of *Gibbonsia* or *Heterostichus*. *G. elegans* was reported to have a fecundity of about 2,300 eggs/female[35]. Fitch and Lavenberg[36] stated that *Gibbonsia* spp. first spawn at 2 years of age, might spawn more than once per year, and have a life expectancy of about 7 years. No survivorship information was available for either genus of kelpfish, so no FH and AEL estimates could be calculated. Daily growth rates of *Gibbonsia* spp. were also unavailable, but estimates for lab-reared larval *H. rostratus*[37] were determined using linear regression as 0.25 mm/day ± 0.013 mm/day (slope ± 1 SE). This growth rate, although not for the correct genus, was substituted for *Gibbonsia* spp. to allow calculation of the ETM for kelpfish.

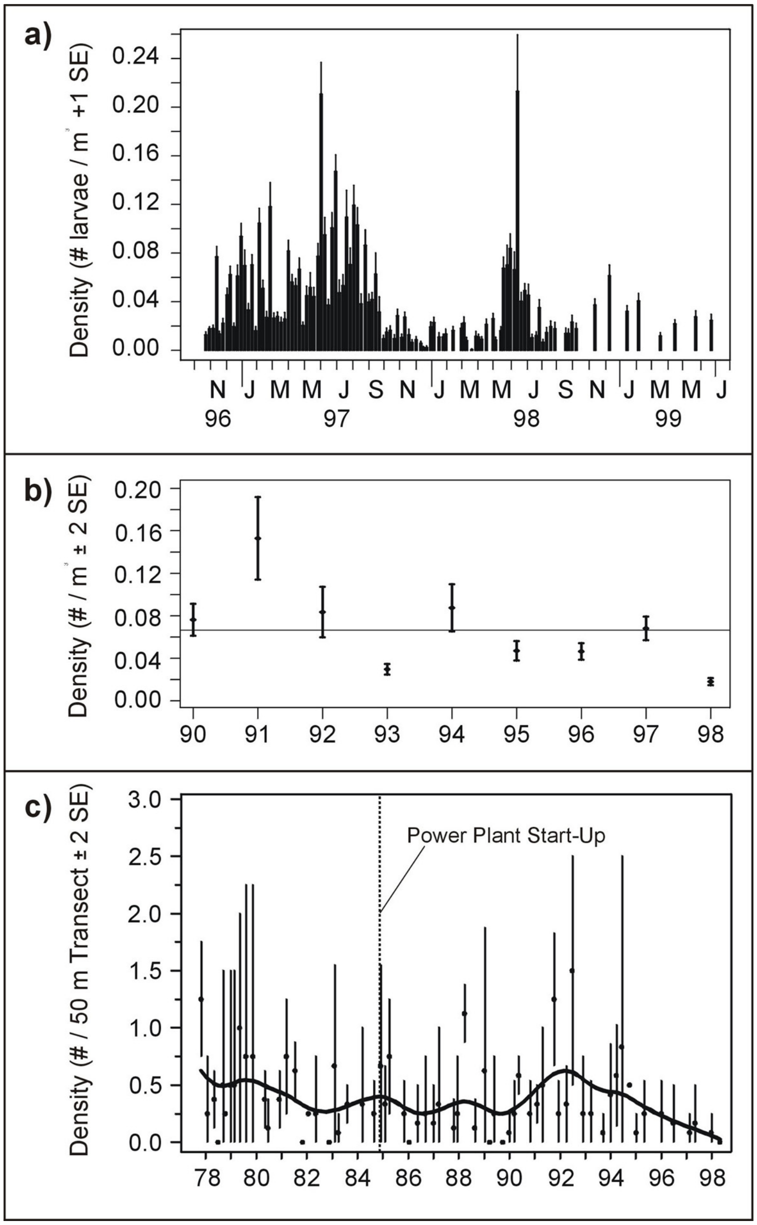

**FIGURE 5. (a)** Weekly mean density of larval kelpfish (#/m$^3$ + 1SE) at the DCPP intake. **(b)** Annual mean density ± 2 SE of larval kelpfish (vertical lines) and grand mean density for all years combined (horizontal line) for the intake cove surface plankton tows. **(c)** Mean density of kelpfish (#/50 m transect ± 2 SE) estimated from SCUBA surveys in an area 700 m south of the DCPP intake cove. Spline smoothing algorithm used to draw the curve through the points.

Kelpfish larvae were present throughout the year in entrainment samples (Fig. 5a). Using estimates of weekly entrainment densities, the estimated numbers of larval kelpfish entrained annually for the two periods, after adjustment to the long-term average intake cove surface plankton tow index, were

October 1996 through September 1997 – 181,000,000 (SE = 4,610,000) larvae, and

October 1997 through September 1998 – 308,000,000 (SE = 15,300,000) larvae.

The monthly PE used in ETM calculations ranged from 0.001 ± 0.002 (± 1 SE (PE)) to a maximum of 0.346 ± 0.189. These larvae were mainly collected in the nearshore area of the grid, and therefore $P_S$ was calculated using only alongshore current movements and not onshore movement as was done for the KGB complex larvae. The values of $P_M$ from both years based on the average larval age at entrainment ranged from 0.294–0.318, and from 0.395–0.410 for the maximum age at entrainment.

*Gibbonsia* spp. are small and cryptic, not commercially or recreationally sought, and almost nothing is known of their trophic role in the coastal ecosystem where they occur. The calculated $P_M$ values cannot be converted into an estimate of adult equivalent loss because nothing is known about the population size or adult density of kelpfish. Thus, we must turn to other sources of information to determine whether entrainment losses constitute an AEI for this taxon. Data from the intake cove surface plankton tows indicate a decline in larval kelpfish abundance from 1995–1998 (Fig. 5b), and the local adult kelpfish abundance appears to be declining from 1993–1998 (Fig. 5c). These local declines combined with ETM results showing up to a 40% reduction of the larvae from an area ca. six to seven times the length of the study grid indicate that the effects on this taxon could be significant and represent a population decline in the vicinity of DCPP.

## CONCLUSION

Three unique assessment models were used to determine the effects of the DCPP cooling-water system on local larval and adult populations. Although AEI was not defined, comparison of the model results in combination with ancillary information on local larval and adult populations of KGB rockfish and clinid kelpfish was helpful in defining the level of impact caused by entrainment at DCPP. The similar results from the three models and stable local populations provide us with high confidence in our determination of no localized impact for this taxa. In the case of clinid kelpfish, withdrawal of about 40% of the available larvae appears to have led to a measurable decrease in the local adult population. It was estimated that the operation of the CWIS at San Onofre Power Plant in California reduced the adult recruitment and adult standing stock in the Southern California Bight by 13% for queenfish and 6% for white croakers[38]. An entrainment rate of 23% by the Wabash River Generating Station was felt to possibly be high enough to impact year-class strength of certain species, yet follow-up studies detected no short-term adverse impacts to the fish community[39].

The DCPP study was unique in having long-term data on abundances of larval and adult fish populations in the vicinity of the plant. The larval data collected from 1990–1998 allowed us to adjust annual entrainment estimates to the long-term average for a species. Entrainment studies are typically done for a period of 1 to 2 years and have an implicit assumption that the data for those years are representative. By adjusting the entrainment estimates to the average larval abundance over a 9-year period, we were able to address the question of sampling in a representative year. The long-term data on adult populations provided context for interpreting the results of our modeling. In the cases of the small, nearshore species that have not been extensively studied, it was the only data available.

Ultimately, the 316(b) demonstration at DCPP did not progress to a formal determination of which effects, if any, could be designated AEIs. Thus, while our approach to defining AEI remains untested, it still shows promise as a way to qualitatively decide if an effect is important and whether it might be considered an adverse environmental effect. To determine this we would have to arbitrarily define a cutoff for AEI (e.g., 40% reduction of larval population) and then combine the interpretation of results from the three approaches as a measure of confidence that the “adverse” effect was either significant or not. If results from the three approaches agreed with each other, then confidence would be high and vice versa. Nevertheless, this definition would likely have been site- or species-specific since much of the context for qualitatively assessing the value of the effects would have to rely on local landings, economics, and population sizes.

## ACKNOWLEDGEMENTS

Grateful thanks go to Pacific Gas and Electric Co. for funding this project, and to its project manager, Ms. Anne Jackson, for her support during this entire project. We also thank the members of the ETWG for their technical guidance and support during this study: Michael Thomas (CCRWQCB), Dr. Greg Cailliet (Moss Landing Marine Laboratories), Drs. Allan Stewart-Oaten and Roger Nisbet (University of California at Santa Barbara), Dr. Pete Raimondi (University of California at Santa Cruz), Deborah Johnston (California Department of Fish and Game), and Anne Jackson and Pat Eckhardt (Pacific Gas and Electric Company). Finally, we acknowledge the staff of Tenera Environmental for all of their time and hard work on the field collection and laboratory processing of the samples. We would also like to thank Mary Nishimoto and an anonymous reviewer for comments that assisted the authors in clarification of certain aspects of this document.
